# The relationship between mindful self-care and maternal-fetal attachment in pregnant women: the parallel mediating effects of positive and negative affect

**DOI:** 10.3389/fpsyt.2025.1672476

**Published:** 2025-09-24

**Authors:** Lika Xu, Xueyi Sun, Yifan Liu, Yujie Chen, Caiyun Wang, Ting Li, Hanjiao Liu, Junfan Wei, Meng Li

**Affiliations:** ^1^ Department of Obstetrics, The First Affiliated Hospital of Zhengzhou University, Zhengzhou, China; ^2^ Department of Histology and Embryology, North Henan Medical College, Xinxiang, China; ^3^ Department of Obstetrics, Zhengzhou Central Hospital Affiliated to Zhengzhou University, Zhengzhou, China; ^4^ Department of Obstetrics, South China Hospital of Shenzhen University, Shenzhen, China; ^5^ Nursing Department, Shenzhen Hospital of Integrated Traditional Chinese and Western Medicine, Shenzhen, China; ^6^ Seventh Clinical Medical College, Guangzhou University of Chinese Medicine, Shenzhen, China; ^7^ Nursing Department, Nanjing Central Hospital, Nanjing, China; ^8^ Nursing Department, The Third People’s Hospital of Henan Province, Zhengzhou, China

**Keywords:** maternal health, mindful, maternal-fetal attachment, self-care, mediating effect, regulation of emotion

## Abstract

**Objective:**

To investigate the relationship between mindful self-care and maternal-fetal attachment (MFA) and parallel mediating effects of positive and negative affect among pregnant women.

**Methods:**

A cross-sectional survey using convenience sampling from June 2025 to July 2025 in six tertiary hospitals in Henan and Guangdong province. A total of 351 pregnant women were recruited and a questionnaire including socio-demographic characteristics, positive and negative affect scale and maternal antenatal attachment scale was used. Descriptive statistics, t-tests, one-way ANOVA, Harman’s single-factor test, Pearson correlation analysis, and parallel mediation analysis were conducted.

**Results:**

Correlation analysis showed that mindful self-care, positive affect, negative affect and MFA were all significantly correlated with each other, respectively. Once positive affect and negative affect were included as mediators, the direct effect of mindful self-care on MFA was no longer significant, suggesting that positive affect and negative affect fully mediated the relationship between mindful self-care and MFA.

**Conclusion:**

Positive and negative affect play parallel mediating roles in the relationship between mindful self-care and MFA, emphasizing the critical role of emotion regulation in developing prenatal MFA and underscore the important value of mindful self-care as a psychological resource during pregnancy, which provides new perspectives for future theoretical models and intervention designs.

## 1 Introduction

Maternal-fetal attachment (MFA) refers to the unique emotional, cognitive, and behavioral bond that a pregnant woman establishes with her unborn child during pregnancy, which reflects the mother’s sense of identity, care, and responsibility for the unborn (1). Recent perspectives view MFA as a dynamic psychological process shaped by maternal perceptions and experiences, beginning early in gestation and manifested through behaviors like touching the belly and preparing for the baby (2). MFA is the emotional bond that connects the mother to the fetus and promotes the physical and mental health and relationship development of the mother and child during the pregnancy and childbearing process. Tahereh Rahimi et al. (3) conducted a study on 220 pregnant women and found that MFA scores were significantly and positively correlated with maternal health behaviors. MFA not only plays an important role in promoting mothers’ health during pregnancy, but also has a significant impact on the mental health of pregnant women. Empirical studies have shown that high attachment was associated with less psychological distress (4) during pregnancy (e.g., depression) and elevated positive emotion and self-efficacy (5) in pregnant women. In addition, high levels of MFA contribute to healthy fetal development and parent-child bonding after birth. A study suggested that MFA predicts postpartum mothers’ quality of care for their infants, including mothers’ emotional availability and mothers’ sensitivity during early mother-infant interactions (1, 6). Another study conducted a three-year longitudinal study of 221 predominantly Black/African American mothers showed that MFA levels were positively correlated with children’s socio-emotional competence at age 3 years, and MFA indirectly affected children’s behavioral functioning and development by influencing maternal parenting stress, suggesting that MFA may have long-term effects on children’s socio-emotional and behavioral functioning (4). A growing body of research has been devoted to exploring the factors influencing MFA. Empirical studies have confirmed that the formation of MFA is significantly influenced by the psychological state of the pregnant woman (e.g., anxiety, depression) and socio-environmental factors (e.g., partner support, experience of health care services). For example, mood disorders have been identified as a major psychological risk factor for interfering with attachment formation (7), while intimate partner support and healthcare interactions have been shown to significantly enhance emotional bonding during pregnancy (8). However, there is still a lack of systematic exploration of the mechanisms of how an individual’s intrinsic psychological resources influence MFA, and in particular, the role that positive psychological traits play in this process is unclear.

Mindful self-care refers to an individual’s ability and behavioral tendency to practice physical, mental, and emotional multidimensional self-care with a mindful attitude - i.e., present-moment awareness, non-judgment, and acceptance (9). Mindful self-care integrates the present-moment awareness of mindfulness with the psychological orientations of acceptance, non-judgment, and self-kindness in self-care. Specifically, mindful self-care not only includes healthy behaviors such as good sleep, nutrition, and physical activity, but also emphasizes the ability to treat oneself with acceptance and kindness in the face of stress, which translates into functional psychological resources (10, 11) that provide individuals with cognitive and emotional regulatory advantages (12). Grzybowski & Brinthaupt’s research further suggests that mindful self-care is closely related to positive self-talk, mental resilience, and is part of a protective psychological mechanism (10). During pregnancy, which is a period of intense physiological and psychological changes, pregnant women are often faced with multiple stressors, including role shifts, physical discomfort, and mood swings (13, 14). As an internal psychological resource, mindful self-care may play a vital role in this context. Higher levels of mindful self-care have been found to be helpful for pregnant women to achieve complete psychological well-being during the COVID-19 pandemic (15). In addition, mindfulness interventions have been shown to be effective in enhancing MFA. However, most studies have focused on the effects of mindfulness interventions, and there is a lack of exploration the role of mindful self-care in fostering MFA in pregnant women. Addressing these gaps is essential to provide insight into the relationship between mindful self-care and MFA, and may provide insights for further refining theoretical frameworks and developing interventions that are effective in enhancing MFA in pregnant women.

Positive affect refers to a positive state of activation in an individual’s subjective experience, such as happiness, energy, confidence, focus and alertness. It is a state of mind in which internal energy is high and with a strong interest in the external world. Negative affect, on the other hand, reflects an individual’s tendency to react emotionally to adverse experiences, such as tension, fear, anxiety, anger and shame. It is a state of subjective distress, avoidance, or defensiveness. These two concepts are derived from David Watson’s Two-Dimensional Model of Affect (16), which posits that positive affect and negative affect are two core dimensions of emotional experience that are not simply opposed but independent of each other. Positive and negative affect are recognized as important psychological variables influencing MFA levels during pregnancy, a highly emotionally sensitive period. Positive affect has been shown to enhance the mother’s emotional engagement and positive behavioral responses to the unborn child (17), in contrast to negative affect, especially anxiety and depressive symptoms, which may undermine the mother’s emotional connection to the unborn child (18). In addition, mindful self-care has been shown to alleviate negative emotions and psychological distress—particularly those related to bodily disconnection—thereby enhancing psychological well-being during pregnancy (12).

According to Frederickson’s Broaden-and-Build Theory (19), positive emotions broaden the scope of an individual’s immediate thinking and action and promote the formation of lasting psychological resources, including cognitive flexibility, emotional resilience, and social connections, enhance cognitive flexibility, behavioral creativity, and facilitate the construction of sustained personal resources such as social connectedness and emotional resilience. This broadened foundation of psychological resources is particularly beneficial during pregnancy, as it allows mothers to engage more deeply in fetal attachment behaviors and emotional coordination (20). Conversely, when positive emotions are low and negative emotions dominate, there may be a depletion of psychological resources, leading to emotional detachment, increased stress responses, and reduced maternal sensitivity-all factors known to inhibit MFA (18, 21). Meanwhile, Hobfoll’s Conservation of Resources (COR) Theory (22) suggests that individuals strive to obtain, retain, and protect psychological resources, and experience stress when these resources are threatened or lost. As a stable internal psychological resource, mindful self-care can alleviate negative emotions and protect resource reserves when individuals feel stress, thereby enhancing emotional availability and commitment to important relationships, including the unborn child. Accumulating evidence underscores that the positive and negative affect of pregnancy plays an important role in shaping MFA: while positive affect fosters warmth, sensitivity, and prenatal bonding behaviors, elevated negative affect may hinder the development of emotional closeness with the fetus (23).

**Figure 1:**
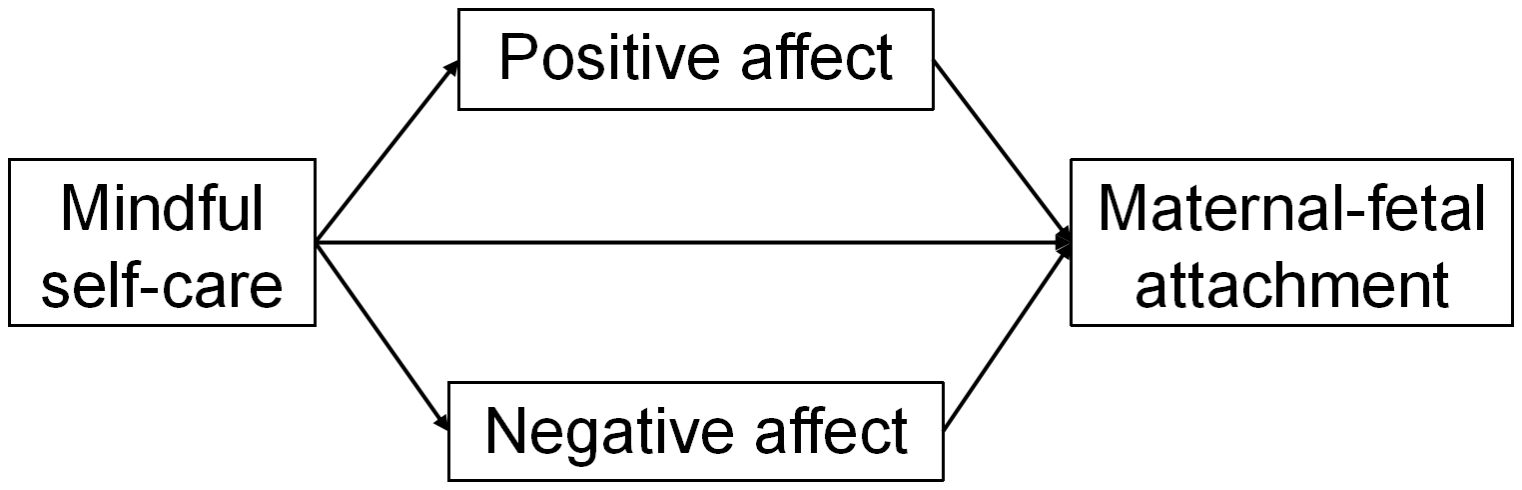
Theoretical framework.

The above empirical and theoretical studies provide clues for exploring the relationship between mindful self-care and MFA and suggest the potential for positive and negative affect to play mediating roles in this relationship. However, there is a research gap in related areas and it is necessary to conduct targeted studies to explore the mechanisms underlying the relationship between mindful self-care and MFA. Filling these gaps will help provide further insights into the relationship between mindful self-care and MFA, and could provide insights for further refinement of the theoretical framework and developing interventions that are effective in enhancing MFA. Therefore, based on the results of existing empirical and theoretical studies and in conjunction with the purpose of the study, we propose the following hypotheses: (1) there is a significant positive correlation between mindful self-care and MFA; (2) positive affect mediates the relationship between mindful self-care and MFA; and (3) negative affect mediates the relationship between mindful self-care and MFA. **Figure 1** illustrated our theoretical framework.

## 2 Methods and materials

### 2.1 Study design

The present study was implemented using the method of convenience sampling and cross-sectional design. We adhered to Strengthening the Reporting of Observational Studies in Epidemiology (STROBE) guidelines and methodology in reports of cross-sectional studies (24).

### 2.2 Sample size

The sample size formula for an observational cross-sectional study was used in this study (25):


$$n={\left(\frac{U_{\alpha /2}\sigma }{\delta }\right)}^{2}={\left(\frac{1.96\times 7.30}{1.468}\right)}^{2}\approx 95$$

where n represents the sample size required to estimate, is the standardized normal deviation corresponding to α= 0.05 and 95% confidence level ( $U_{\alpha /2}$ = 1.96, for two-tailed), *σ* represents the expected value of standard deviation in the population (*σ* = 7.30 from existing literature (18)), and *δ* represents the acceptable margin of error for the mean ( δ =3 from existing literature (18)). Considering the 20% invalid questionnaires, the sample size for this study should be at least 119 (26).

### 2.3 Participants and data collection

We conducted a cross-sectional survey using convenience sampling. Data collection was conducted from June 2025 to July2025 in six tertiary hospitals in Henan and Guangdong province. The data collection process strictly complied with the core principles of data acquisition and privacy protection (27, 28).

We followed the following procedures for data collection. Following approval from hospital administrators, researchers distributed paper questionnaires to participants in quiet, private settings (e.g., conference rooms or consultation areas) to ensure confidentiality. The inclusion criteria for the participants were as follows (1) pregnancy diagnosed by a clinician, (2) age of at least 18 years, and (3) voluntary participation in this study. The exclusion criteria for the participants were (1) the pregnant women had a previous history of psychiatric illness; (2) the pregnant women suffered from severe pregnancy comorbidities such as diabetes mellitus, gestational hypertension, or eclampsia; and (3) the pregnant women were unable to complete the communication using Mandarin or had dyslexia, which made it difficult for them to complete the questionnaires independently.

To enhance the reliability and accuracy of the data, multiple quality assurance strategies were adopted. Prior to the commencement of the study, the research team conducted several group discussions to establish unified guidelines for interpreting key study concepts, thereby minimizing the risk of misinterpretation. All researchers responsible for administering the questionnaires received standardized professional training to ensure consistent implementation. Upon completion of each questionnaire, researchers reviewed them immediately to verify completeness. During the data entry phase, two trained researchers independently managed the input of paper-based data into the digital system. Cross-verification was conducted to detect and promptly resolve any discrepancies. All eligible participants received both verbal and written explanations of the study’s purpose, their rights as participants, and the confidentiality measures in place. Written informed consent was obtained from each participant prior to their participation. The study protocol received ethical approval from the institutional review board, and all data were securely stored with restricted access limited to the research team. A total of 500 participants were invited to participate in this study, and 351 valid questionnaires were collected, resulting in an effective response rate of 70.2%.

### 2.4 Measures

#### 2.4.1 Socio-demographic characteristics questionnaire

The questionnaire was designed by the researcher and the data collected were objective, including age, educational attainment, monthly per person family income, residence location, pregnancy stage, history of adverse pregnancy experiences, pregnancy complications.

#### 2.4.2 Brief-mindful self-care scale

This scale is a brief version of the Mindful Self-care Scale, designed to assess the degree of mindful self-care. The Brief Mindful Self-care Scale was originally developed and validated in English by Cook-Cottone et al. (29). The Chinese version was translated by Yang et al. (30). The scale includes six subscales: mindful relaxation, physical care, self-compassion and goals, supportive relationships, supportive structure, and mindfulness awareness. These six dimensions consist of 24 items, each rated on a 5-point Likert scale. The scale ranges from 1 to 5, representing: never, rarely, sometimes, often, and always. The total score ranges from 24 to 120, with higher scores indicating a higher level of mindful self-care. The Chinese version of the scale demonstrates acceptable reliability and validity, with Cronbach’s α for each dimension ranging from 0.850 to 0.933 (30).

#### 2.4.3 Positive and negative affect scale

The PANAS was developed by Watson and Clark in the United States in 1988 to assess respondents’ mood in the last 1–2 weeks (16). The scale consists of two dimensions with a total of 20 items, of which items 1, 3, 5, 9, 10, 12, 14, 16, 17, and 19 were used to assess positive affect; whereas items 2, 4, 6, 7, 8, 11, 13, 15, 18, and 20 were used to assess negative affect. Each item was rated on a 5-point Likert scale from 1 (very slightly or not at all) to 5 (extremely). The English version of PANAS demonstrates good reliability and validity, and it has been translated into several languages, including Dutch (31), Arabic (32), and so on, all of which show good cross-cultural stability. The Chinese version of PANAS was adapted into Chinese and imported into China by Li Huang et al. The results showed that the Cronbach’s α coefficients of all the items in the Chinese version PANAS were 0.82, and the Cronbach’s α coefficients of positive and negative affect were 0.85 and 0.83 respectively, demonstrating good reliability and validity.

#### 2.4.4 Maternal antenatal attachment scale

The MAAS was developed in 1993 by Australian psychologist Condon, J. T (33). to assess the emotional attachment status of mothers to their unborn fetus during pregnancy. The MAAS consists of 19 items, administered on a self-reporting format, that reflect the psychological and behavioral attachment of pregnant women to their fetus during pregnancy. All items are rated on a five-point Likert scale (e.g., 1=Almost never, 5=Almost always). Total scores ranged from 19-95, with higher total scores indicating stronger emotional attachment of the mother to the fetus. The English version of the MAAS showed favorable reliability and validity during the application process. The Chinese version of the MAAS was introduced by Ge Nie and Hongxia Fan (34), who Chineseized, translated, expert reviewed, distributed questionnaires and analyzed reliability and validity of the scale, with a sample size of 545 pregnant women. The results showed that the Chinese version of the scale exhibited acceptable reliability and validity, with the Cronbach’s α was 0.77.

### 2.5 Statistical analysis

All statistical analyses were conducted using SPSS version 27.0 (SPSS Inc., Chicago, IL, USA). Initially, descriptive statistics were employed to characterize the socio-demographic characteristics of the pregnant women. For categorical data, frequencies and percentages were used (35), while continuous data were presented as ( $\bar{x}$ ± s). Independent samples t-tests and one-way ANOVA were employed to assess differences in MAAS score among pregnant women with different socio-demographic characteristics. To assess the potential impact of common method bias, Harman’s single-factor test was conducted, given that the measurements were based on self-reported scales (36, 37). Next, Pearson correlation analysis were carried out to explore the bivariate relationships between mindful self-care, positive affect, negative affect, and MFA. Lastly, a mediation model was tested using the bias-corrected bootstrapping method with PROCESS 4.1 in SPSS 27.0 (38). To examine the parallel mediating effects of positive affect and negative affect on the relationship between mindful self-care and MFA among pregnant women, Mode 4 of PROCESS was applied for the mediation analysis. Our proposed model hypothesized that mindful self-care (X) enhances positive affect (M1) and reduces negative affect (M2), which subsequently leads to an increase in MFA (Y). A 95% bias-corrected confidence interval (CI) was generated using 5000 bootstrapped re-samples. A CI excluding zero was considered indicative of a significant mediation effect (39). In addition, the model was controlled for covariates, including age, educational attainment, monthly per person family income, residence location, pregnancy stage, history of adverse pregnancy experiences and pregnancy complications. This study was ethically approved by the Medical Ethics Committee of the Third People’s Hospital of Henan Province (approval no. 2025SZSYLCYJ0602). The investigation was conducted in accordance with the Declaration of Helsinki.

## 3 Results

### 3.1 Socio-demographic characteristics of participants

The mean age of 351 pregnant women recruited was (30.98 ± 4.027). In the present study, most of the participants had a bachelor’s degree (48.718%), monthly per capita household income was 3000–5000 RMB (43.305%), lived in cities (72.934%), were in the third trimester of pregnancy (96.866%), did not have a history of adverse pregnancy (70.940%), and did not have complications during pregnancy (75.499%). Among the socio-demographic characteristics, participants’ varying age, educational attainment, per person monthly family income, residence location, and pregnancy complications had an impact on MAAS scores. See **Table 1** for details.

**Table 1:** The level of MAAS by different socio-demographic characteristics (N = 351).

Variables	*n* (%)	MAAS		
		M ± SD	*t or F*	*P*
Age	351 (100.000)	75.73 ± 6.577	0.294	<0.001
Educational attainment			16.460	<0.001
Below college education	51 (14.529)	74.59 ± 7.144		
Junior college’s degree	114 (32.479)	72.93 ± 6.962		
Bachelor’s degree	171 (48.718)	78.04 ± 5.541		
Graduate degree	15 (4.273)	74.67 ± 0.488		
Monthly per person family income (in CNY)			3.936	0.020
<3000	65 (18.519)	75.74 ± 7.753		
3000~5000	152 (43.305)	74.68 ± 6.141		
>5000	134 (38.177)	76.84 ± 6.577		
Residence location			11.416	<0.001
Countryside	68 (19.373)	74.35 ± 6.032		
Town	27 (7.692)	81.15 ± 3.538		
City	256 (72.934)	75.53 ± 6.699		
Pregnancy stage			1.045	0.307
Second trimester (13 weeks to 27 weeks of pregnancy)	11 (3.133)	77.72 ± 1.133		
Third trimester (28 weeks to delivery)	340 (96.866)	75.67 ± 6.651		
Have you had any adverse pregnancy experiences, such as miscarriage, difficult labor, etc.?			0.256	0.613
Yes	102 (29.060)	76.01 ± 5.570		
No	249 (70.940)	75.62 ± 6.955		
Do you have any pregnancy complications, such as gestational hypertension, gestational diabetes, etc.?			-5.606	<0.001
Yes	86 (24.501)	72.42 ± 5.780		
No	265 (75.499)	76.81 ± 6.464		

n, number; %, percentage; M, mean; SD, standard deviation. RMB to USD exchange rate in mid-2025 was approximately 1 USD ≈ 7.2 CNY.

### 3.2 Common method bias

In this study, the impact of common method bias was controlled by using anonymous responses and concealing variable names. The Harman’s single-factor test showed that the variance explained by the first eigenvalue was 27.747%, which was < 40%, indicating that there is no serious common method bias in this study (40).

### 3.3 Correlation study

**Figure 2:**
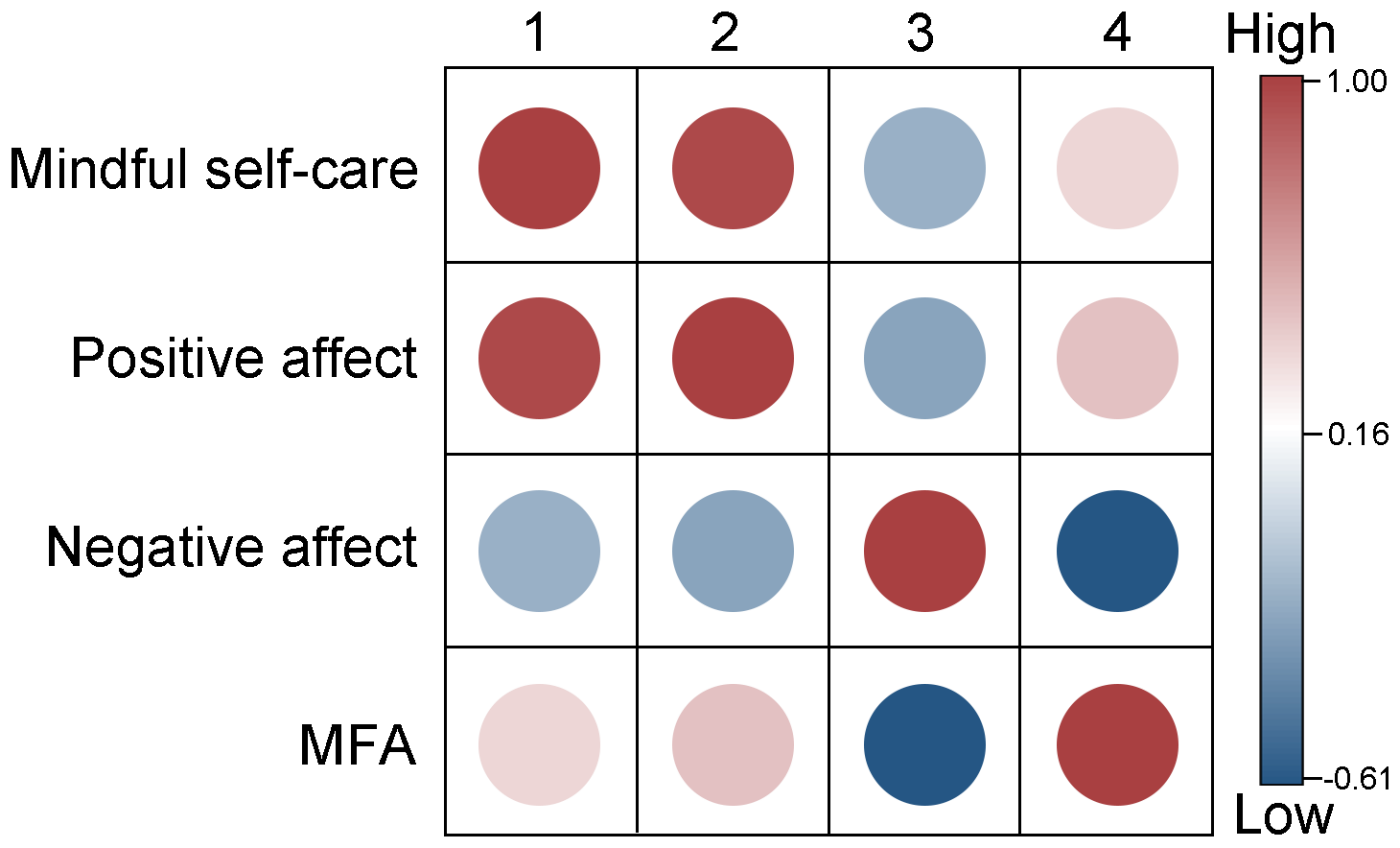
Heatmap of correlations among mindful self-care, positive affect, negative affect and MFA. MFA, maternal-fetal attachment.

**Table 2** provided the means, standard deviations, and correlations among the variables. The results showed that mindful self-care was positively correlated with positive affect (*r* = 0.954, *P* < 0.001) and MFA (*r* = 0.410, *P* < 0.001), and negatively correlated with negative affect (*r* = -0.151, *P* < 0.001). Positive affect was negatively correlated with negative affect (*r* = -0.228, *P* < 0.001) and positively correlated with MFA (*r* = 0.465, *P* < 0.001). Negative affect was negatively correlated with MFA (*r* = -0.601, *P* < 0.001). See **Figure 2** for the correlation heatmap.

**Table 2:** Correlations among mindful self-care, positive affect, negative affect and MFA.

Variables	M	SD	1	2	3	4
1.Mindful self-care	82.003	15.605	1			
2.Positive affect	34.160	7.222	0.954***	1		
3.Negative affect	33.330	8.048	-0.151***	-0.228***	1	
4.MFA	75.732	6.577	0.410***	0.465***	-0.601***	1

***P < 0.001; M, mean; SD, standard deviation.

### 3.4 Parallel mediation model analysis

A parallel mediation analysis was performed to examine the role of positive affect and negative affect as parallel mediators in the indirect relationship between mindful self-care and MFA among pregnant women, using Model 4 in the SPSS PROCESS macro. Mindful self-care and MFA were specified as the independent (X) and dependent (Y) variables, respectively. Positive affect (M1) and negative affect (M2) were treated as mediators, while age, educational attainment, monthly per person family income, residence location, pregnancy stage, history of adverse pregnancy experiences, and pregnancy complications were included as covariates.

**Figure 3:**
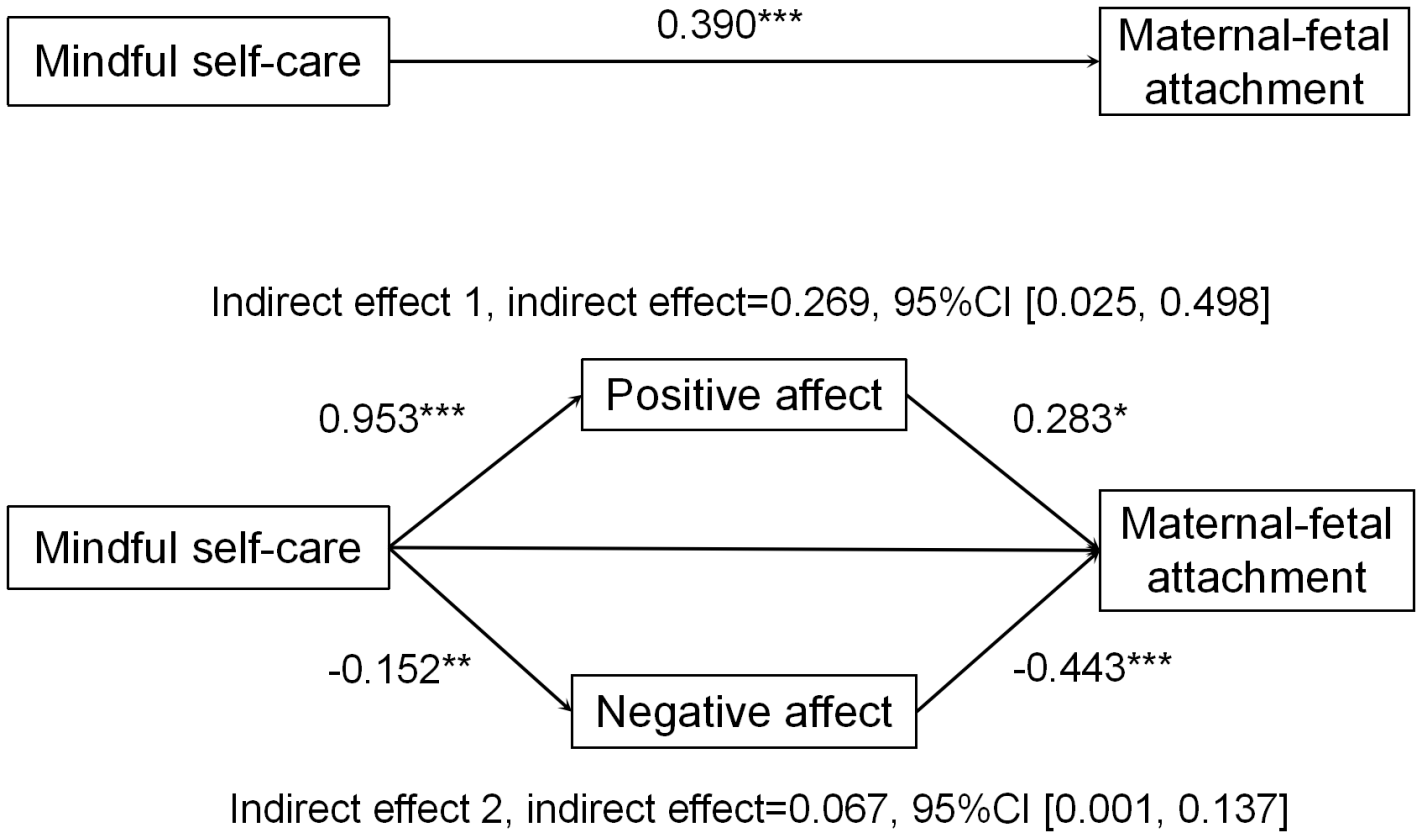
Standardized regression coefficients in the model. *P < 0.05, **P < 0.01, ***P < 0.001. Age, educational attainment, monthly per person family income, residence location, pregnancy stage, history of adverse pregnancy and pregnancy complications were treated as covariates in the parallel mediation model. Path coefficients are standardized.

As demonstrated in **Table 3** and **Figure 3**, the results of the mediation analysis conducted with the PROCESS macro indicate that, after adjusting for covariates, the total effect of mindful self-care on MFA was significant (*β* = 0.390, *P*<0.001). After including positive and negative affect as mediators, the direct effect of mindful self-care on MFA was no longer significant, suggesting that positive affect and negative affect fully mediated the relationship between mindful self-care and MFA.

**Table 3:** Total, direct, and indirect effects of the mediation model.

Effect	Product of coefficients		Bootstrapping 95% CI	
	Point estimate	Boot SE	Lower	Upper
Total effect of MS on MFA	0.164***	0.020	0.125	0.204
Direct effect of MS onMFA	0.023	0.053	-0.825	0.128
Total indirect effect of MS on MFA	0.142***	0.051	0.036	0.567
Indirect 1: MS→PA→MFA	0.114***	0.051	0.025	0.498
Indirect 2: MS→NA→MFA	0.028***	0.015	0.001	0.137

SE, standard error*;* CI, confidence interval; ***P < 0.001. Age, educational attainment, monthly per person family income, residence location, pregnancy stage, history of adverse pregnancy, pregnancy complications were treated as covariates in the chain mediation model. All paths are expressed as unstandardized.

Bootstrap estimation procedure (n = 5000) indicated that the indirect effects for this model were statistically significant (indirect effect = 0.142, SE = 0.051, 95% CI =[0.036, 0.567], **Table 3**). The indirect effects were generated through two paths: Path 1 consisting of mindful self-care ⟶ positive affect ⟶ MFA (indirect effect=0.114, 95% CI =[0.025, 0.498]), confirming the significant mediating role of positive affect. Path 2 consisting of mindful self-care ⟶ negative affect ⟶ MFA (indirect effect=0.028, 95% CI [0.001, 0.137]), confirming the significant mediating role of negative affect.

## 4 Discussion

This study investigated the impact of mindful self-care on MFA among a sample of pregnant women, as well as the parallel mediating roles played by positive affect and negative affect. The results showed a positive correlation between mindful self-care and MFA, and this association was achieved through the parallel mediating roles played by positive affect and negative affect. By exploring the effects of mindful self-care on MFA in pregnant women and its underlying mechanisms, this study may provide new perspectives for understanding MFA in pregnant women.

The results of our study showed that age, per person monthly family income and pregnancy complications were the influencing factors of MFA in pregnant women, which were consistent with previous studies (7, 41, 42). This implies that healthcare professionals should offer greater emotional and prenatal support to younger pregnant women, and financial assistance to those from low-income households, and more physical and psychological support for pregnant women with pregnancy complications.

In this study, we found that pregnant women with a bachelor’s degree had the highest level of MFA, followed by those with less than a junior college’s degree and those with a graduate degree, and pregnant women with a junior college’s degree had the lowest level of MFA. This result conflicts with the current view that higher education is associated with stronger MFA (7, 41). Although the small sample size of women with graduate degrees limits the statistical reliability of this finding, it still holds exploratory value. This suggests that the impact of education on MFA may not be simply linear, and higher academic achievement does not necessarily equate to stronger mother-infant attachment. One possible explanation is that educational attainment is not an isolated factor but interacts with emotional states (43), social support (44), and maternal experience (41, 45). In certain social environments, pregnant women with graduate degrees may devote much of their energy to academic studies, careers, or professional training, and this professional pressure and time management issues may cause them to neglect the importance of emotional well-being and prenatal education during pregnancy, while less educated pregnant women may rely on more emotional support and social interactions. Therefore, social support and prenatal health education (46) may be more important for enhancing MFA connection than simply enhancing educational attainment. Notably, pregnancy stage may also play a role in this relationship. In our study, nearly all participants (96.9%) were in the third trimester, while the second-trimester subgroup included only 11 participants—all of whom had relatively lower educational levels (junior college’s degree). Although no statistically significant differences in MFA scores were observed between different stages of pregnancy in our study, the small sample size in the mid-pregnancy group limited the reliability of this comparison. Previous research has shown that MFA tends to increase as pregnancy progresses, due to enhanced fetal movements, emotional readiness, and bonding behaviors (2). Therefore, the high MFA observed among women with bachelor’s degrees—most of whom were in the third trimester—may reflect not only educational influences, but also the cumulative emotional development that occurs later in pregnancy. Future studies with a more balanced distribution of gestational stages are needed to clarify the potential interaction between education and pregnancy stage on MFA.

In our study, pregnant women residing in towns exhibited the highest levels of MFA. Although this subgroup comprised only 27 participants (7.7% of the total sample), and generalization should therefore be made cautiously, the finding offers a potentially meaningful insight into the influence of residential environments on prenatal emotional bonding. Several unique social and environmental characteristics of town settings may help explain this observation. Towns often offer a moderate pace of life and relatively accessible yet less overstressed healthcare services—conditions that differ markedly from the high-pressure, fast-paced, and socially fragmented nature of cities, or the limited healthcare infrastructure in rural areas. These balanced conditions may create a more supportive environment for fostering maternal emotional well-being and fetal attachment. This interpretation aligns with findings by Lindgren et al. (47), who observed higher MFA levels and more favorable health behaviors in pregnant women from smaller urban communities. In addition, social support is one of the core factors in the formation of MFA. Compared to social isolation and neighborhood alienation, which are common in cities, pregnant women in towns are more likely to receive emotional support from their families, neighborhoods, and communities, thus contributing to MFA formation (48). And while there are sufficient human bonds in the countryside, pregnant women are less likely to develop stable emotional connections due to the lack of systematic prenatal education and medical support (49). Thus, this middle ground advantage in education and resources may be an important reason why pregnant women in towns perform better in MFA than those in cities and the countryside. While further studies with larger and more balanced samples are needed to confirm these patterns, our findings contribute valuable preliminary evidence suggesting that residential environment may play a role in shaping MFA.

In the present study, we found a strong positive association between mindful self-care and MFA, which aligns with previous research findings that suggest mindful self-care practices play a critical role in enhancing maternal emotional bonding during pregnancy (12). Prior empirical findings have demonstrated that intentional self-care and pregnancy embodiment reduce distress and foster connection with the fetus, particularly under high-stress contexts such as the COVID-19 pandemic.

Additionally, the effect of mindful self-care on MFA was no longer significant when the positive affect and negative affect were introduced, indicating that positive affect and negative affect fully mediated the association between mindful self-care and MFA. This result suggests that mindful self-care regulates emotional states, which in turn indirectly affects their emotional connection to the fetus. Our findings support Cook-Cottone’s theory (9), which emphasizes that mindful self-care behaviors are a resourceful regulatory strategy with emotion regulation functions that can help pregnant women enhance self-awareness, alleviate stress reactions, and develop self-compassion, thereby activating positive emotions and buffering the effects of negative emotions. These findings are further supported by previous empirical research, which found that mindful self-care could influence pregnant women’s distress and well-being during pregnancy (12).

From a theoretical perspective, these findings match well with Watson’s Two-Dimensional Model of Affect (16), which hypothesizes that positive and negative affect are separate dimensions of emotional experience rather than opposing ends of a single continuum. This model provides a key framework for interpreting the observed parallel mediation model: positive and negative affect are distinct but simultaneous mediators, reflecting the double pathway through which mindful self-care influences MFA. Mindful self-care does not merely contribute to the production of positive emotional states; it simultaneously serves to reduce distress and buffer negative emotions, thereby facilitating emotional connection.

The positive affect pathway is particularly consistent with Frederickson’s Broaden-and-Build Theory (19), which suggests that positive emotions expand an individual’s momentary thought-action reservoir and build lasting personal resources such as resilience and social connections. In the context of pregnancy, increased positive emotions expressed as joy, gratitude, calmness, or hope may stimulate intimate prenatal behaviors (e.g., talking to the baby, imagining future parenthood) that deepen emotional engagement with the fetus (50, 51). Furthermore, previous studies have shown that positive emotional states in pregnant women are associated with increased MFA and maternal sensitivity in the postpartum period (52, 53), emphasizing the importance of this pathway.

In contrast, the negative affect pathway emphasizes the harmful effects of negative emotions (e.g., anxiety, fear, or anger) on maternal bonding. Elevated negative affect is associated with diminished emotional engagement and impaired prenatal attachment (54). Our findings extend these observations by demonstrating that mindful self-care significantly reduced negative emotions, thereby removing emotional barriers to MFA formation. This is consistent with previous research showing that mindfulness practice reduces pregnancy-related distress and enhances mothers’ emotional regulation and sensitivity to their unborn child (55).

Furthermore, these results can be interpreted within the framework of Hobfoll’s COR theory (22), which hypothesizes that individuals strive to acquire and maintain psychological resources and experience stress when these resources are threatened. Mindful self-care, as a personal psychological resource, protects individuals from emotional depletion and fosters psychological resilience, both of which contribute to maintaining connection with the unborn child during stressful periods of pregnancy.

The present study contributes to a deeper understanding of the relationship between mindful self-care and MFA by exploring the underlying psychological mechanisms by which mindful self-care may influence MFA. Mindful self-care appears to influence MFA primarily by enhancing positive affect and alleviating negative affect, rather than playing a direct role, thus highlighting the critical role of emotion regulation as a potential pathway for strengthening the maternal-fetal bond. In addition, this study provides a valuable reference point for designing interventions for maternal mental health. The observed emotional pathways suggest that promoting mindful self-care practices may help to improve the emotional state of pregnant women, thereby creating a more favorable environment for the development of MFA. To enhance clinical utility, our findings support integrating brief mindful self-care training into routine prenatal care settings, such as during obstetric visits, prenatal classes, or community health programs. Interventions may include structured group sessions led by trained nurses, midwives, or mental health professionals, focusing on mindful, emotional awareness, and self-compassion. Such programs may be particularly beneficial for emotionally vulnerable pregnant women, providing them with practical tools for regulating emotions and strengthening prenatal bonds. Future research could further validate the effectiveness of incorporating mindful self-care strategies into prenatal support programs as a complementary approach to promoting mothers’ emotional well-being and early attachment, especially for women who are emotionally vulnerable during pregnancy.

There are several limitations to this study. Firstly, the cross-sectional research design used in this study prevents causality from being inferred and long-term effects between variables from being discussed. Therefore, the results of the current study need to be interpreted with caution and future longitudinal studies should be conducted to determine how the relationships between these variables change over time. Secondly, this study employed convenience sampling by selecting six city hospitals, limiting the generalizability of the findings. Potential biases may stem from geographic location, cultural background, and socioeconomic position, as participants may have access to hospital care and share similar social contexts. Additionally, self-selection bias may exist, as more proactive or engaged women may be more inclined to participate. These factors could influence observed MFA levels, necessitating consideration of more diverse and representative samples in future research. Thirdly, although we adjusted for certain covariates, other unmeasured factors—such as partnership support, psychiatric history, lifestyle habits, personality traits, pregnancy planning, and socioeconomic stress—may also influence MFA and should be considered in future research. In addition, the sample was heavily weighted toward women in late pregnancy (96.9%), limiting the ability to discern differences across pregnancy stages and reducing the generalizability of the findings to early pregnancy. Lastly, the use of self-report measures may introduce bias; future studies should consider using clinical interviews or observational tools to enhance measurement validity.

## 5 Conclusion

The present study examined the relationship between mindful self-care and MFA in pregnant women and confirmed the parallel mediating role of positive and negative affect in it. The findings showed that mindful self-care did not directly affect MFA, but exerted its influence exclusively through the emotional pathway, i.e., enhancing positive emotions and decreasing negative emotions. These results emphasize the critical role of emotion regulation in the construction of prenatal MFA and underscore the important value of mindful self-care as a psychological resource during pregnancy. By identifying both positive and negative affect as key mediators, this study provides new perspectives for future theoretical models and intervention designs aimed at promoting mother’ emotional well-being and fostering early MFA.

## Data Availability

The raw data supporting the conclusions of this article will be made available by the authors, without undue reservation.
